# Bristol General Hospital

**Published:** 1913-06-07

**Authors:** 


### Bristol General Hospital.

### TWO INTERESTING PRESENTATIONS.

On Monday, last week, a very pleasant ceremony was held at the Bristol General Hospital to mark the occasion of the retirement of two of the senior sisters. Sister Thomas, who has charge of the three medical wards —Artisan, Thomas, and Powell—has been actively connected with the work of the hospital for twenty-five years, and Sister Fry has for many years been in charge of the surgical wards—Fry and Dod—with a total period of twenty years to her credit. There were present Mr. Herbert M. Baker, chairman of the hospital, Mr. Francis Sturge, vice-chairman; Miss Densham, matron; Mr. Duncan Wood, senior house surgeon; Mr. W. Thwaites, secretary, and many members of the house committee, ladies' committee, honorary, resident, and nursing staff, and other friends. After a few introductory remarks by the Chairman, Mr. Herbert M. Baker, the Senior Honorary Physician, Dr. Michell Clarke paid a warm tribute of admiration to the exceptionally valuable work which had been done by Sister Thomas during her long and useful career. Dr. J. Lacy Firth, in a very humorous speech, followed with a eulogistic account of the virtues of Sister Fry, and then many valuable and appropriate presentations were made. Sister Thomas received a cheque for £117, a silver teapot, and an address from the committee and past and present staff, a purse containing £12 from past nurses, silver spoons and a clock from the sisters, and an escritoire from the present nurses. Sister Fry was presented with a cheque for £105, a silver teapot, and an address from the committee and past and present staff, a purse containing £5 from past nurses, a Thermos flask and a clock from the sisters, and a dinner service and rug from the present nurses. The presentations were made by the Chairman, by Mrs. Thwaites, the former matron, and by Miss Densham. After the recipients had suitably responded, tea was served, and so ended a particularly pleasant episode which will not soon be forgotten.

